# Post-Stroke Asymmetric Prurigo Nodularis Responding to Dupilumab Treatment: A Case Report

**DOI:** 10.3390/brainsci12050605

**Published:** 2022-05-05

**Authors:** Ilaria Sammarra, Luigi Bennardo, Eugenio Provenzano, Cataldo Patruno, Steven Paul Nisticò

**Affiliations:** 1 Department of Medical and Surgical Sciences, Magna Graecia University, 88100 Catanzaro, Italy; ilaria.sammarra@studenti.unicz.it; 2 Department of Health Sciences, Magna Graecia University, 88100 Catanzaro, Italy; luigi.bennardo@studenti.unicz.it (L.B.); cataldo.patruno@unicz.it (C.P.); nistico@unicz.it (S.P.N.); 3 Unit of Dermatology, Mariano Santo Hospital, 87100 Cosenza, Italy

**Keywords:** post-stroke prurigo nodularis, dupilumab, anti-IL-4/13

## Abstract

Prurigo nodularis (PN) is a dermatological condition characterized by nodular hyperkeratotic lesions mainly on the legs and arms. Asymmetrical PN is a rare dermatological condition often associated with paralysis and stroke. In this paper, we present the case of a 77-year-old woman who developed post-ictal PN which responded to dupilumab, an anti-interleukin-4/13 drug approved for the management of AD, with an extreme reduction in itch sensation. Dupilumab and other therapies reducing Th2 inflammation may, in the future, become an alternative treatment for post-ictal pruritus/PN nonresponding to traditional therapies. Of course, larger studies will be necessary to confirm our case’s findings.

## 1. Introduction

Neuropathic itch (NI) recognizes multiple etiologies deriving from the peripheral nervous system [1]. Central NI is a rarer case of pruritus following several brain diseases, such as tumors, ischemic stroke, multiple sclerosis, and abscesses. Post-stroke NI may originate from a lesion in the brainstem, thalamus, and somatosensorial cortex, affecting the descending spinothalamic tract. Lateral medullary syndrome is reported as a cause of post-stroke itch, generally predisposing to trigeminal trophic syndrome (TTS) [2]. No therapies have been approved for NI, but some improvements have been recorded using antidepressants, sodium channel blockers, and gabapentinoids [1]. Prurigo nodularis (PN) is a complex dermatological condition characterized by itchy nodules that usually appear on the legs and arms, usually excoriated due to the patient’s scratches [3]. The etiology of the condition is unknown, and it has been associated with various conditions, such as liver disease, autoimmune conditions, and atopic dermatitis (AD) [4]. Asymmetrical PN, although rare, is a recognized entity usually associated with paralysis and strokes. In some rare cases, the underlying etiology may remain unknown [5]. Various treatments such as steroids, thalidomide, cryotherapy, and immunosuppressants have been proposed, with unsatisfactory results [6,7]. This report describes the case of a patient presenting NI with PN following a lateral medullary ischemic stroke and responding to anti-interleukin-4 (IL-4)/interleukin-13 (IL-13) monoclonal therapy.

## 2. Case Presentation

**Figure 1:**
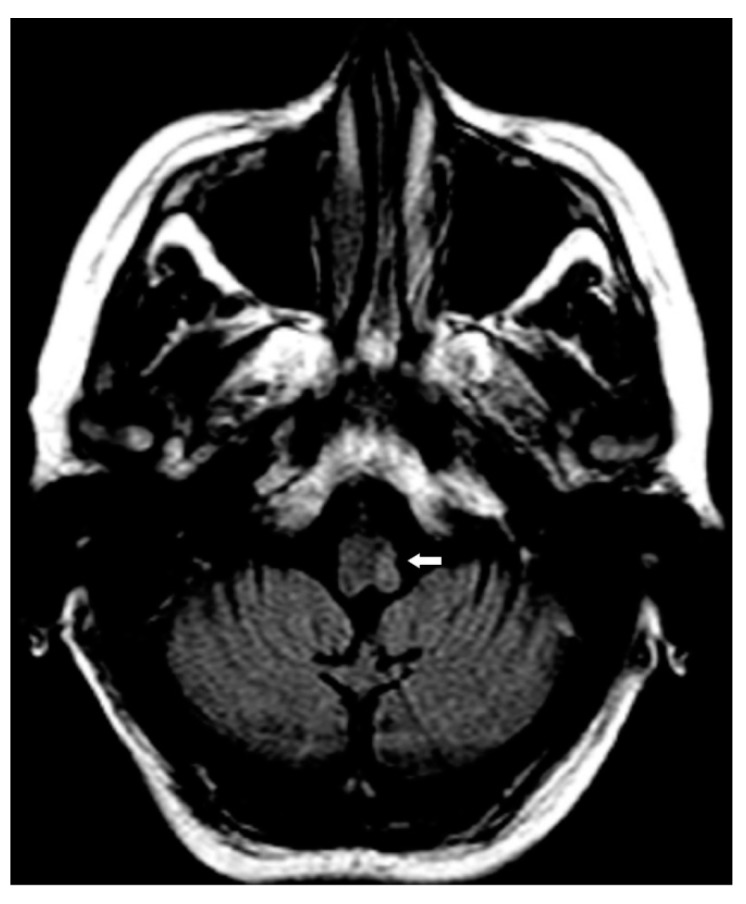
An axial FLAIR-weighted MRI image showing a hyperintense area (white arrow) in the left lateral medulla, suggestive of infarct.

**Figure 2:**
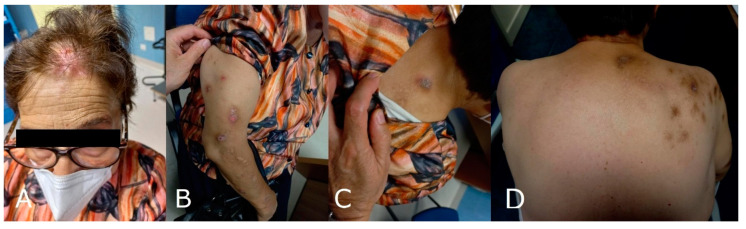
Multiple hyperkeratotic nodules on the right side of the head (**A**), the right upper limb (**B**), and the right upper trunk (**C**,**D**) before dupilumab treatment.

**Figure 3:**
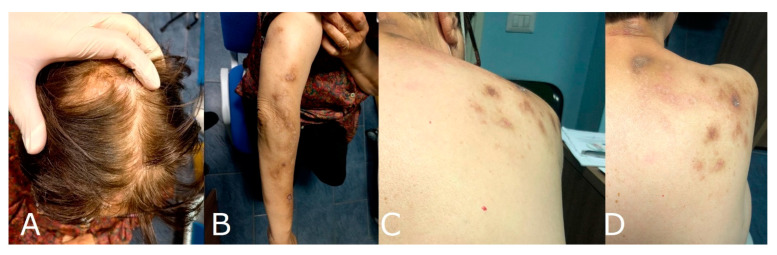
Multiple hyperkeratotic nodules on the right side of the head (**A**), the right upper limb (**B**), and the right upper trunk (**C**,**D**) after 16 weeks of dupilumab treatment; a reduction in inflammation and the number/thickness of lesions may be seen.

A 77-year-old woman presented to our dermatological center with a 2-year history of severe itch associated with thick nodules in the right side of the head, trunk, and right upper limb. At the cutaneous examination, the left side of the body was completely spared. The patient judged her itch intensity as 9/10 on a numerical rating scale (NRS) [8]. The patient was previously affected by a mild form of AD, treated with good control with topical steroids and emollients. She reported no history of other previous skin diseases. At the age of 75 years, she presented an ischemic stroke, manifesting with left hemiparesis, lower left facial weakness, hypoesthesia in the right side of the body, dysarthria, dysphagia, gait instability, brisk reflexes in the left limbs, and left extensor plantar response. The brain MRI localized the stroke in the left lateral medulla, configuring Opalski syndrome [9,10] (Figure 1). Concomitantly with the other symptoms, she manifested a severe itch restricted to the right side of the head, extending to the right upper limb and trunk in the following weeks. After two months, she presented multiple hyperkeratotic nodules in the same area affected by pruritus. In the following 2 years, she assumed different types of medication for her itch, such as oral and topical corticosteroids, antihistaminic drugs, and amitriptyline, but she showed no improvement (Figure 2). To better understand the nature of her nodules, we performed a skin biopsy, and the histological exam demonstrated the presence of thick compact orthohyperkeratosis, irregular epidermal hyperplasia, focal parakeratosis, hypergranulosis, and a superficial infiltrate of mainly lymphocytes and macrophages. According to the clinical manifestations, type of stroke lesion, and pathology result, post-stroke PN was diagnosed [2]. Given the absence of response to traditionally proposed therapies, and the contemporary presence of AD, we started a biologic treatment with dupilumab, a monoclonal therapy targeting the IL-4R alpha subunit shared by the IL-4 and IL-13 receptor complexes. At the first administration, she received 600 mg followed by 300 mg every two weeks as maintenance treatment. After 16 weeks, she manifested a clinical improvement in itch intensity, judged as 4/10 on the NRS, associated with a reduction in PN lesions (Figure 3). No adverse reactions were reported.

## 3. Discussion

Pruritus is a rare post-stroke symptom whose underlying pathophysiology overlaps with the most common post-stroke pain [11]. NI might derive from damage of the spinothalamic tract, thalamus, or somatosensory cortex [1]. Specifically, ischemic lesions of the lateral medulla could configure classic Wallenberg or Opalski syndrome based on the presence of ipsilateral hemiparesis and contralateral sensory impairment. In both cases, the spinothalamic tract results in being injured, configuring ipsilateral or contralateral hypoesthesia [10,12]. TSS, characterized by unilateral itch-induced ulceration and lower face anesthesia, is a well-described condition associated with about 21% of cases with vertebra-basilar stroke damaging the trigeminal pathway [2,13]. Given the spinothalamic tract involvement, an NI affecting the same areas of sensory impairment might be a rarer manifestation of lateral medullary syndrome [13]. In the areas involved in chronic itch, a skin biopsy could demonstrate characteristic neuropathic changes, such as PN. This above-mentioned manifestation is generally caused by peripheral NI, as in polyneuropathies. PN exhibits dermal, interstitial, and perivascular inflammation, perpetuating the vicious circle of repeated itching and scratching through multiple mediators, such as histamine, prostaglandins, IL-31, and IL-4 [14]. Although no treatment is currently approved, several medications have been tested for NI, both topical and systemic. Capsaicin cream or topical anesthetics are possible choices. Anyway, oral drugs, including anti-seizure drugs, such as carbamazepine, and antidepressants, such as amitriptyline, are the main options for NI treatment. In cases of refractory itch, thalidomide could be a possible choice, especially when pruritus is associated with PN, but this treatment’s main limiting factor is its severe adverse reactions [1,2]. The fully human monoclonal antibody dupilumab is currently approved to treat AD. AD is a common inflammatory skin disease characterized by skin and systemic inflammation, and barrier dysfunction [15]. This condition is associated with a dysregulation of the adaptive and innate immune responses and an immunoglobulin E-mediated, systemic Th2 response to environmental antigens [16]. Th2 lymphocytes, when stimulated, release pro-inflammatory cytokines, such as IL-4, 13, and 31, that seem implicated in the pathogenesis of both AD and PN [17,18]. Given its selective action against the IL-4 pathway, and the good therapeutic outcome in all subtypes of AD [19,20], this biologic therapy has been explored as a possible medication for PN, exhibiting promising results in a phase 3 trial [14]. This report described the first case of post-stroke PN responding to dupilumab treatment. Therefore, this anti-IL-4 monoclonal antibody might represent a consistent alternative therapy for PN resistant to other drugs with no significant collateral effects. A similar case, with the onset of PN following stroke and involving the areas of paralysis and dysesthesia, has been reported by Chu and colleagues [21]. In this type of patient, the itch–scratch cycle may be of major importance in the spreading of PN in the involved areas [22]. For this reason, a therapy acting on itch inflammatory mediators, such as an anti-IL-31 drug or, as in our case, an anti-IL-4 or IL-13 treatment, may tremendously improve the patient’s quality of life [23].

In conclusion, we may state that dupilumab showed its effectiveness in the management of stroke-associated PN and that it, as with other biologic drugs acting on Th2 inflammation, may retain an effect on various types of neuropathic itches. Of course, prospective studies and clinical trials will be necessary in order to confirm the result obtained in our patient.

## Data Availability

Data are available from the corresponding author upon reasonable request.
